# Decreased low-frequency amplitude of the right caudate nucleus in patients with chronic schizophrenia treated with risperidone combined with clozapine

**DOI:** 10.3389/fpsyt.2025.1630499

**Published:** 2025-08-29

**Authors:** Xinyue Chen, Lixing Lei, Hangyu Li, Ying Li, Shiji Peng, Kaike Liao, Rui Yu, Nian Liu

**Affiliations:** Department of Radiology, Affiliated Hospital of North Sichuan Medical College, Nanchong, China

**Keywords:** schizophrenia, risperidone, clozapine, resting-state magnetic resonance imaging, amplitude of low-frequency fluctuation

## Abstract

**Backgound:**

While risperidone and clozapine monotherapies have been linked to distinct neuroimaging profiles in chronic schizophrenia, the combined effects of these treatments on brain function remain unclear. This study aimed to compare spontaneous neural activity between patients receiving risperidone monotherapy and those undergoing risperidone-clozapine combination therapy, and to investigate how these neural alterations relate to clinical symptomatology.

**Methods:**

This study enrolled 28 patients with chronic schizophrenia who had been treated with long-term risperidone monotherapy (RT-SZ), 40 patients receiving long-term combination therapy with risperidone and clozapine (RCT-SZ), and 30 healthy controls (HCs) comparable in sex, age, and educational level. Resting-state functional magnetic resonance imaging (rs-fMRI) was utilized for acquiring neural data, and amplitude of low-frequency fluctuation (ALFF) was computed to examine activity in different brain regions. Group comparisons were conducted using analyses of covariance (ANCOVAs) with age, sex, and educational level as covariates, followed by *post-hoc* testing. Partial correlation analyses were performed to examine associations between ALFF alterations and clinical symptoms or cognitive performance.

**Results:**

Both the RT-SZ and RCT-SZ groups exhibited significantly reduced ALFF in the bilateral lingual gyrus and right middle occipital gyrus, along with raised ALFF in the right caudate nucleus and right medial superior frontal gyrus, relative to HCs. Additionally, the RT-SZ group showed elevated ALFF in the left caudate nucleus, while the RCT-SZ group demonstrated decreased ALFF in the left postcentral gyrus. Notably, the RCT-SZ group exhibited reduced ALFF in the right caudate nucleus compared to the RT-SZ group. Partial correlation analysis revealed a positive trend between ALFF in the left lingual gyrus and measures of attention and information processing speed among chronic schizophrenia patients.

**Conclusions:**

This exploratory analysis observed a more pronounced decrease in right caudate nucleus ALFF in the combination therapy group compared to the risperidone monotherapy group. The observed differences in neural activity patterns provide preliminary neuroimaging clues suggesting potential distinct neural effects between long-term monotherapy and combination therapy in chronic schizophrenia, and may offer new directions for exploring neuroimaging explanations for the combination therapy.

## 1 Introduction

Schizophrenia is a debilitating, chronic psychiatric disorder defined by a complex array of symptoms, including hallucinations, delusions, cognitive impairments, and social dysfunction (1). Chronic schizophrenia typically refers to patients who have experienced the illness for over five years and continue to exhibit persistent symptoms despite treatment efforts (2). Antipsychotic drugs, especially atypical second-generation antipsychotics, are essential for the pharmacological management for schizophrenia (3). Among these, risperidone and clozapine are widely prescribed and have been shown to effectively reduce psychotic symptoms and decrease relapse rates (4). Given the lifelong nature of treatment for individuals affected by this condition (5), the emerging field of psychoradiology (6) has increasingly focused on understanding how prolonged antipsychotic exposure influences brain structure and function. Prior neuroimaging efforts have probed the long-term impacts of monotherapy with either risperidone or clozapine by comparing their impact on brain structure (4, 7–9). For example, comparative structural MRI studies have found that clozapine monotherapy is associated with greater reductions in gray matter in the bilateral prefrontal cortex and left cuneus, but with relatively less atrophy in the left ventral temporal lobe compared to risperidone treatment alone (8). Similarly, a comparative analysis of white matter fiber change has demonstrated that clozapine-treated patients exhibit more widespread white matter microstructural disruption compared to the risperidone-treated group (9), involving decreased FA values in bilateral anterior thalamic radiation, bilateral inferior fronto-occipital fasciculus, bilateral inferior longitudinal fasciculus, left corticospinal tract, left uncinate fasciculus, right arcuate fasciculus, the splenium and the genu of the corpus callosum, right cingulum-hippocampus pathway. Another study on white matter structural networks revealed that clozapine-treated patients exhibited relatively severe alterations in nodal and connectivity features compared to risperidone-treated patient (4). Significant associations were observed between changes in global network metrics and cognitive performance levels (4). A previous study employing voxel-mirrored homotopic connectivity-based analyses has shown that long-term olanzapine treatment can normalize default node, sensory, and motor network functional connectivity (7b). However, the majority of these investigations have focused exclusively on monotherapy, leaving the neurophysiological consequences of combination therapy largely unexplored.

Our prior work has focused on structural brain changes in schizophrenia patients receiving either risperidone or clozapine monotherapy (4, 8, 9). In clinical practice, however, risperidone-clozapine combination therapy is increasingly adopted for patients with insufficient response to monotherapy and may offer enhanced therapeutic efficacy (10, 11). Risperidone and clozapine target different neurotransmitter systems and exhibit complementary pharmacological actions. Several studies suggest that their co-administration may produce synergistic or interaction effects through mechanisms such as receptor competition (12), altered pharmacokinetics (13), and distinct molecular binding profiles (14). Despite this, the potential neural consequences of combined antipsychotic therapy have yet to be clarified through functional imaging studies, contributing to a gap in objective neuroimaging evidence to inform treatment strategies.

This study aimed to determine whether long-term combination therapy with risperidone and clozapine produces different changes in spontaneous brain activity compared to risperidone monotherapy in patients with chronic schizophrenia. Resting-state functional magnetic resonance imaging (rs-fMRI), a widely utilized and reproducible technique, enables assessment of intrinsic brain activity without requiring task performance (15), was used for this purpose. Although functional connectivity analysis assesses inter-regional synchrony (16) and regional homogeneity assesses local synchrony (17), which are crucial for understanding brain network mechanisms and local coordination, and dynamic functional connectivity performance reveals temporal variability (18), these indicators reflect more the spatial or temporal organization patterns of neuronal activity rather than the intensity level of the activity itself. The fractional amplitude of low-frequency fluctuation (fALFF) reduces the influence of physiological noise by standardizing the entire frequency spectrum (19). However, standard ALFF has been widely adopted and validated in previous studies on the pathophysiology and drug treatment of schizophrenia, and is directly associated with the intensity of spontaneous brain activity (20–22). Schizophrenia patients have been found to exhibit abnormal ALFF in multiple brain regions (21, 23). More importantly, multiple studies have reported that changes in ALFF values are associated with the severity of patients’ clinical symptoms and their response to antipsychotic drug treatment (24–27). These findings support ALFF as an effective biomarker for assessing treatment-related neurobiological effects. Since the primary focus of the study was on changes in the intensity of spontaneous neural activity in the brain, ALFF was selected as the main indicator in this study to provide preliminary evidence of differences in spontaneous brain function and fill research gaps. This also lays the foundation for future studies to integrate more complex indicators to comprehensively analyze the multi-level effects of drug treatment on brain networks. At the same time, to minimize confounding effects, we carefully matched study participants based on demographic and clinical factors, and defined long-term treatment as continuous medication use for six months or longer (28, 29), in line with previous findings that neurometabolic effects of antipsychotics typically stabilize by this duration (30). We hypothesized that combination therapy would produce unique alterations in ALFF compared to risperidone monotherapy, reflecting distinct neurobiological mechanisms. The findings are expected to contribute to a deeper insight into the neuroimaging correlates of combination antipsychotic treatment and support its clinical rationale.

## 2 Method and materials

### 2.1 Participants

The study was conducted in accordance with the Declaration of Helsinki. The Medical Ethics Committee of the Affiliated Hospital of North Sichuan Medical College gave study approval (2022ER504-1). All subjects or their legal guardians gave written informed consent.

Eligibility criteria for patient inclusion were: (1) a confirmed diagnosis of schizophrenia as per the Structured Clinical Interview for the DSM-5; (2) aged 18–60 years; (3) right-handed; (4) illness duration of at least five years, verified through patient self-report, family member interviews, medical documentation, and additional corroborative sources; and (5) continuous treatment for no less than six months with a stable dose of either risperidone alone or in combination with clozapine, with no significant changes to the medication regimen during this period.

Healthy control participants (HCs) were recruited via local community advertisements. For HCs, criteria for inclusion were: (1) no personal history of psychiatric or neurological disorders as determined by screening with the SCID-Non-Patient Version; (2) Han Chinese ethnicity; (3) right-handed; and (4) aged 18–60 years. Controls were closely matched to the schizophrenia groups based on age, sex, and education years,

Exclusion criteria included: (1) MRI contraindications such as metallic implants, pacemakers, or severe claustrophobia; (2) history of substance or alcohol abuse; (3) concurrent severe physical illness or central nervous system disorders; (4) recent significant infections, surgical procedures, or a history of traumatic brain injury; and (5) current pregnancy or breastfeeding. All MRI scans were reviewed by an experienced radiologist to rule out subjects with structural brain abnormalities.

Participants were recruited from two psychiatric institutions in Guang’an, Sichuan Province, China: Wusheng Bashu Psychiatric Hospital and Wusheng Ruikang Psychiatric Specialized Hospital. This study used G*power 3.1 software (31) to calculate the required sample size, with an effect size of 0.4 and a significance level of 0.05. The calculation results showed that in order to achieve a statistical power of 0.8, a total of 64 participants were needed for the three groups, and 98 participants were actually recruited. Patients diagnosed with chronic schizophrenia were assigned to one of two treatment groups based on their ongoing medication regimen: the risperidone monotherapy group (RT-SZ) or the risperidone combined with clozapine group (RCT-SZ). All patients had not changed the type of antipsychotic medication within the 6 months prior to enrollment and maintained a stable treatment dose (as confirmed by the attending physician that there were no clinically significant adjustments to the dose) to ensure consistency in the medication regimen and dose among enrolled patients. Dosages of antipsychotic medications were standardized by conversion to chlorpromazine (CPZ) equivalents (32). Based on the criteria outlined above, 98 participants were enrolled in this study, comprising three groups: 40 patients with chronic schizophrenia receiving long-term combination therapy with risperidone and clozapine, including 9 women and 31 men with a mean age of (38.98 ± 9.11) years, 28 patients with chronic schizophrenia treated with long-term risperidone monotherapy, consisting of 10 females and 18 males, with a mean age of (38.39 ± 10.22) years; and 30 healthy controls (HCs), including 18 males and 12 females, with a mean age of (38.67 ± 9.46) years.

### 2.2 Clinical symptoms and cognitive evaluation

Symptom severity and cognitive functioning were evaluated using two standardized instruments: the Positive and Negative Syndrome Scale (PANSS) (33) and the Brief Assessment of Cognition in Schizophrenia (BACS) (34), respectively. The BACS battery includes seven subtests targeting various cognitive domains: List Learning test, Digit Sequencing, Token Movement Task, Category Fluency and Word Fluency tests, Symbol Coding, and the Tower of London test. These tests collectively assess verbal memory, working memory, motor speed, verbal fluency, attention and information processing speed, and executive functioning.

### 2.3 Data acquisition

Neuroimaging was conducted using a 3.0T magnetic resonance scanner (Discovery 750, GE Healthcare, Milwaukee, Wisconsin). Participants remained awake and motionless with closed eyes during imaging. High-resolution three-dimensional structural images were captured with a T1-weighted sequence of repetition time (TR) = 7.1 ms, echo time (TE) = 2.7 ms, flip angle (FA) = 9°, voxel resolution = 1 mm³, field of view (FOV) = 256 × 256 mm², slice thickness = 1 mm, and 188 contiguous sagittal slices. The following rs-fMRI sequence parameters were used for scanning: TR = 2500 ms, TE = 28 ms, FOV = 192 × 192 mm², FA = 68°, voxel size = 3 × 3 × 3 mm³, matrix = 64 × 64, slice thickness = 3 mm, slice gap = 0.6 mm, 40 transverse slices, and 250 time points for a total scan duration of 625 seconds.

### 2.4 Data preprocessing and ALFF computation

The rs-fMRI data were preprocessed using the DPARSFA v5.4 toolbox (https://rfmri.org/DPARSF). The initial 10 time points were removed to enable stabilization of the signal. All other images were subjected to slice timing correction and realignment. Participants exhibiting translation or rotation exceeding 2.5 mm or 2.5°, respectively, were not included. Functional images were coregistered to individual T1-weighted anatomical images. T1 images were then normalized to the Montreal Neurological Institute (MNI) standard space with the DARTEL algorithm. Linear trends were removed, anda Gaussian kernel with a full width at half maximum (FWHM) of 4 mm was utilized for spatial smoothing. To control for confounding variables, the regressing out of unwanted white matter signals, cerebrospinal fluid, and head motion (modeled using the Friston 24-parameter model) was performed. After preprocessing, the ALFF was calculated for each voxel using a bandpass filter set at 0.01–0.08 Hz. To normalize the data distribution for group-level comparisons, ALFF values were transformed using Fisher’s z-transformation.

### 2.5 Statistical analysis

R software was utilized for all analysis. P < 0.05 threshold was utilized for significance. Normally distributed continuous data were compared with one-way analyses of variance (ANOVAs) with subsequent Bonferroni corrections. Non-normally distributed data were analyzed using the Mann-Whitney U test or Kruskal-Wallis test, as applicable. Chi-square tests were applied for categorical data. Normally distributed descriptive data are shown as mean ± standard deviation, while non-normally distributed are given as medians (Q1-Q3).

Group comparisons of ALFF values were conducted with analysis of covariance (ANCOVA), with sex, age, and educational level included as covariates, implemented in the DPABI software package. A statistical mask was generated from the ANCOVA results, and *post hoc* comparisons between groups were conducted within this mask using the least significant difference (LSD) method (35). To control for multiple comparisons, a Gaussian random field (GRF) correction was applied (voxel-level P < 0.001, cluster-level P < 0.05, two-tailed). Partial correlation analyses were subsequently applied to assess relationships between ALFF values in significantly different brain regions and clinical variables, including illness duration, medication dosage, PANSS scores, and BACS subscale scores. Spearman’s partial correlations were utilized for non-normally distributed data, using the same covariates. This analysis involved correlation tests between 7 brain regions and 13 clinical indicators. Bonferroni correction was used to reduce the false positive rate of multiple comparisons, with a threshold set at α level 0.05/13/7 (36). Correlations were considered statistically significant only when the original P value was <0.000549.

## 3 Results

### 3.1 Demographic characteristics

There is no significant differences detected among the three groups in terms of age, sex distribution, or years of education. Relative to the HCs, both RT-SZ and RCT-SZ patients exhibited significantly reduced scores in the BACS total scale, as well as in specific cognitive domains including verbal memory, verbal fluency, motor speed, working memory, attention and information processing speed, and executive functioning. In contrast, scores on the PANSS—including positive and negative, as well as general psychopathology subscales and total scores—were markedly raised in both patient groups compared to HCs. Notably, a marked difference in antipsychotic dosage between the RT-SZ and RCT-SZ groups was observed. However, no differences were evident when comparing the two patient groups with respect to illness duration, PANSS total and subscale scores, or BACS total and subscale scores. The demographic characteristics and cognitive assessment outcomes are detailed in **Table 1**.

**Table 1:** Demographic and clinical information of the subjects.

Characteristic	RCT-SZ (n=40)	RT-SZ (n=28)	HC (n=30)	*P* value	*Post hoc* analysis (Bonferroni corrected)		
					RCT-SZ VS RT-SZ	RCT-SZ VS HC	RT-SZ VS HC
Age/years	38.98±9.11^#^	38.39 ± 10.22	38.67 ± 9.46	0.969^a^	1.000	1.000	1.000
Sex (M/F)	31/9	18/10	18/12	0.255^b^	—	—	—
Education/years	8.39 ± 2.47	7.89 ± 3.06	9.47 ± 4.03	0.156^a^	1.000	0.490	0.190
Illness duration/years	12.5 (8,19)^&^	11 (7.5,13)	—	0.285^c^	—	—	—
Medication dosage (chlorpromazine equivalent)	262.5 (237.5,465.63)	200 (200,400)	—	0.034^c^	—	—	—
PANSS positive symptom	9 (7,13)	8 (11.5, 15)	7 (7,7)	<0.001^d^	0.146	<0.001	<0.001
PANSS negative symptom	10.5 (9,12.75)	11.5 (9, 13.75)	7 (7,7)	<0.001^d^	0.285	<0.001	<0.001
General psychopathology symptom	24 (21,28.5)	26.5 (23.25,32)	16 (16,17.25)	<0.001^d^	0.108	<0.001	<0.001
PANSS total score	42.5 (37.25, 53.75)	50 (41.5,62.5)	31 (30,32)	<0.001^d^	0.146	<0.001	<0.001
BACS total score	135.43 ± 47.17	138.89 ± 56.10	231.17 ± 42.43	<0.001^a^	1.000	<0.001	<0.001
Verbal memory	16.25 ± 9.56	18.71 ± 12.37	38.13 ± 13.04	<0.001^a^	1.000	<0.001	<0.001
Working memory	14 (4,20)	16 (5,20)	24 (21.5,27)	<0.001^d^	1.000	<0.001	<0.001
Verbal fluency	17 ± 8.18	16.14 ± 9.17	27.17 ± 7.79	<0.001^a^	1.000	<0.001	<0.001
Executive function	12 (4,15.75)	11 (1.5,16)	19 (15,21)	<0.001^d^	1.000	<0.001	0.001
Attention and processing speed	16.38 ± 10.79	18 ± 13.23	45.2 ± 19.64	<0.001^a^	0.933	<0.001	<0.001
Motor speed	68 (51,76)	66 (52.25,79.5)	80 (75.5,88)	<0.001^d^	1.000	<0.001	0.021

^#^ Values are presented as mean ± SD; ^&^Values are presented as median (first quartile, third quartile). ^a^one-way analysis of variance tests. *Post-hoc* t-test (Bonferroni corrected, P < 0.05); ^b^chi-square test; ^c^Mann-Whitney U test. P < 0.05 is considered significant; ^d^Kruskal-WallisH-test. *Post-hoc* t-test (Bonferroni corrected, P < 0.05). HC, healthy controls; RCT-SZ,combined treatment schizophrenia group; RT-SZ, risperidone-treated schizophrenia patients. PANSS, Positive and Negative Syndrome Scale; BACS, Brief Assessment of Cognition in Schizophrenia.

### 3.2 ALFF comparisons

**Figure 1:**
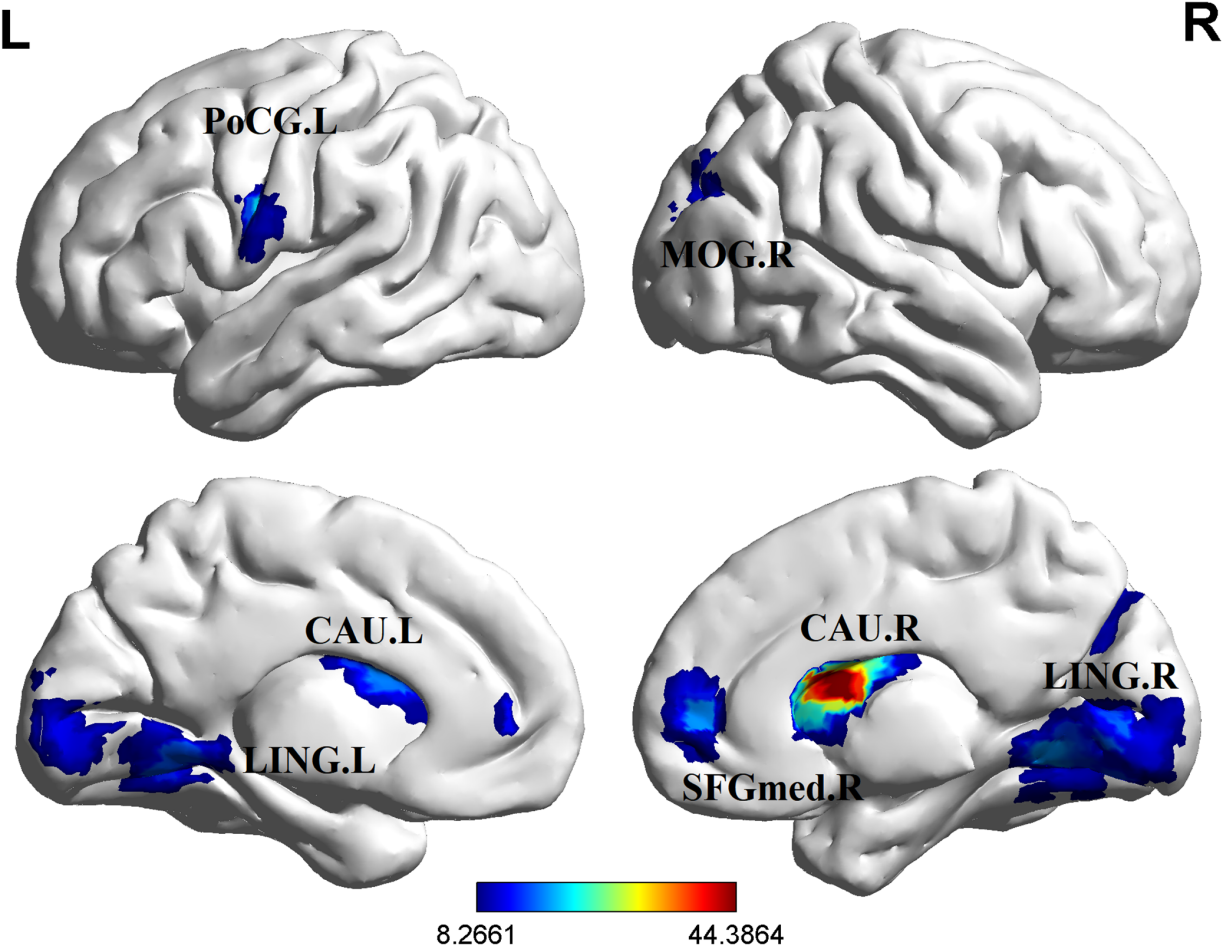
Brain regions with significant differences in ALFF values among the three groups (GRF, P<0.001 for voxel level, P<0.05 for cluster level, two-tailed). L, left; R, right; CAU.L, the left caudate nucleus; CAU.R, the right caudate nucleus; LING.L, the left lingual gyrus; LING.R, the right lingual gyrus; MOG.R, the right middle occipital gyrus; PoCG.L, the left postcentral gyrus; SFGmed.R, the right medial superior frontal gyrus.

**Figure 2:**
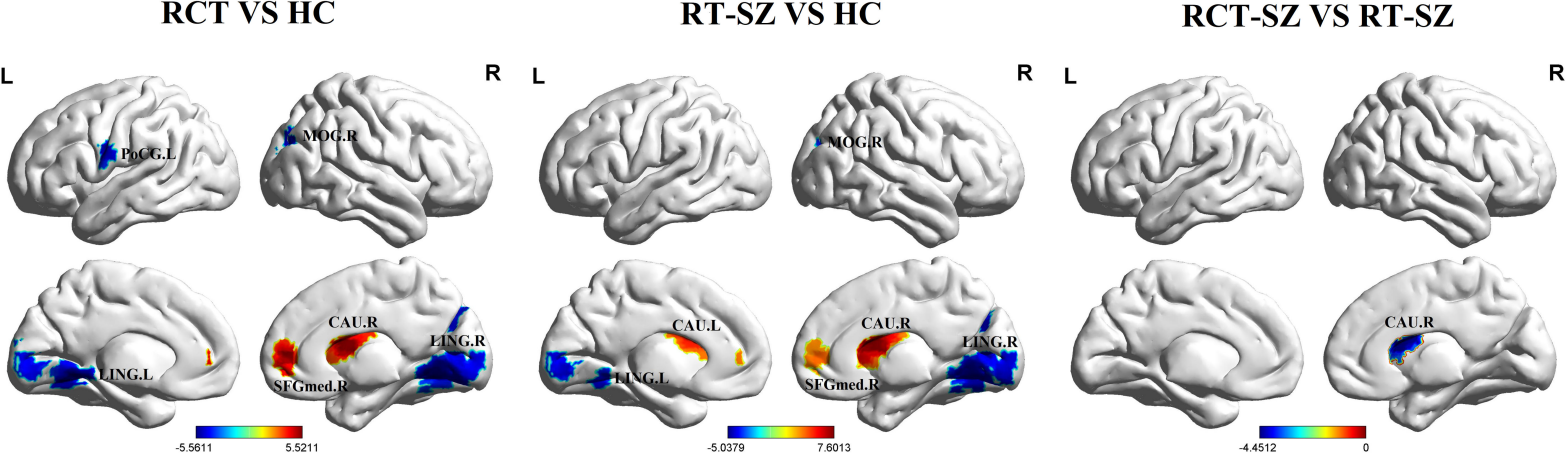
Brain regions with significant differences in ALFF values in *post hoc* tests (GRF, P<0.001 for voxel level, P<0.05 for cluster level, two-tailed). RCT-SZ, combined treatment schizophrenia group; RT-SZ, risperidone-treated schizophrenia group; HC, healthy controls. L, left; R, right; CAU.L, the left caudate nucleus; CAU.R, the right caudate nucleus; LING.L, the left lingual gyrus; LING.R, the right lingual gyrus; MOG.R, the right middle occipital gyrus; PoCG.L, the left postcentral gyrus; SFGmed.R, the right medial superior frontal gyrus.

Significant group differences in ALFF were detected across several brain regions, including the bilateral lingual gyrus, bilateral caudate nucleus, right middle occipital gyrus, right medial superior frontal gyrus, and left postcentral gyrus among these three groups of study participants (**Table 2**, **Figure 1**). Relative to the HCs, RCT-SZ patients showed reduced ALFF in the bilateral lingual gyrus, the right middle occipital gyrus and the left postcentral gyrus, together with increased ALFF in the right caudate nucleus, the right medial superior frontal gyrus (**Table 3**, **Figure 2**). Relative to HCs, RT-SZ patients showed reduced ALFF in the bilateral lingual gyrus, the right middle occipital gyrus, together with increased ALFF in the bilateral caudate nucleus and the right medial superior frontal gyrus (**Table 3**, **Figure 2**). Relative to RT-SZ patients, RCT-SZ patients showed reduced ALFF in the right caudate nucleus (**Table 3**, **Figure 2**).

**Table 2:** Brain regions with significant differences in ALFF between the three groups.

Brain region	Cluster size	F value	MNI coordinate		
			x	y	z
Lingual_R	361	17.966	15	-54	-6
Caudate_R	115	44.386	12	9	15
Lingual_L	77	15.793	-18	-60	-9
Occipital_Mid_R	75	13.944	27	-78	15
Frontal_Sup_Medial_R	43	17.704	6	51	6
Caudate_L	29	17.251	-12	9	21
Postcentral_L	29	20.485	-54	-6	24

ALFF, amplitude of low-frequency fluctuation; L, left; R, right; MNI: Montreal Neurological Institute. Lingual_R, the right lingual gyrus; Caudate_R, the right caudate nucleus; Lingual_L, the left lingual gyrus; Occipital_Mid_R, the right middle occipital gyrus; Frontal_Sup_ Medial_R, the right medial superior frontal gyrus; Caudate_L, the left caudate nucleus; Postcentral_L, the left postcentral gyrus.

**Table 3:** Brain regions with significant differences in ALFF in *post hoc* tests.

Brain region	Cluster size	t value	MNI coordinate		
			x	y	z
RCT-SZ>HC					
Caudate_R	45	5.521	12	9	15
Frontal_Sup_Medial_R	41	5.239	6	51	6
RCT-SZ<HC					
Lingual_R	344	-5.358	15	-54	-6
Lingual_L	77	-5.429	-18	-60	-9
Occipital_Mid_R	53	-4.718	27	-78	15
Postcentral_L	28	-5.561	-54	-6	21
RT-SZ>HC					
Caudate_R	113	7.601	12	9	15
Caudate_L	29	5.285	-12	9	21
Frontal_Sup_Medial_R	20	4.205	6	51	6
RT-SZ<HC					
Lingual_R	131	-4.634	24	-66	-3
Occipital_Mid_R	45	-4.601	33	-72	15
Lingual_L	16	-4.122	-21	-57	-9
RCT-SZ<RT-SZ					
Caudate_R	26	-4.451	9	9	15

ALFF, amplitude of low-frequency fluctuation; HC, healthy controls; RCT-SZ, combined treatment schizophrenia group; RT-SZ, risperidone-treated schizophrenia group. L, left; R, right; MNI: Montreal Neurological Institute. Caudate_R, the right caudate nucleus; Frontal_Sup_Medial_R, the right medial superior frontal gyrus; Lingual_R, the right lingual gyrus; Lingual_L, the left lingual gyrus; Occipital_Mid_R, the right middle occipital gyrus; Caudate_L, the left caudate nucleus.

### 3.3 Correlation analyses

In the uncorrected analysis, partial correlation analysis demonstrated a marked positive relationship between ALFF in the left lingual gyrus and the attention and information processing speed in chronic schizophrenia (*r* = 0.261, P = 0.035, uncorrected). In contrast, illness duration was negatively correlated with ALFF values in the right lingual gyrus (*r* = -0.339, P = 0.006, uncorrected), left lingual gyrus (*r* = -0.27, P = 0.03, uncorrected), and left caudate nucleus (*r* = -0.295, P = 0.017, uncorrected) (**Table 4**). No significant correlations were identified between ALFF in the right caudate nucleus, right middle occipital gyrus, right medial superior frontal gyrus, or left postcentral gyrus and clinical variables such as illness duration, medication dosage, or PANSS and BACS scores (**Table 4**). After controlling for age, gender, and educational level, partial correlation analysis did not reveal any significant correlations between ALFF values in any brain region and clinical indicators after Bonferroni correction.

**Table 4:** Correlation of ALFF alterations with medication dose, disease duration and symptoms in chronic schizophrenia.

Brian regions	Lingual_R		Caudate_R		Lingual_L		Occipital_Mid_R		Frontal_Sup_Medial_R		Caudate_L		Postcentral_L	
	*r*	P	*r*	P	*r*	P	*r*	P	*r*	P	*r*	P	*r*	P
Illness duration/years	-0.339	0.006**	-0.171	0.173	-0.27	0.03*	-0.098	0.44	0.223	0.074	-0.295	0.017*	-0.196	0.118
CPZ	-0.174	0.166	-0.239	0.055	-0.068	0.591	-0.022	0.863	0.13	0.304	0.001	0.996	-0.132	0.296
PANSS positive symptom	0.013	0.916	0.023	0.859	-0.034	0.79	-0.074	0.56	0.045	0.722	0.066	0.604	-0.029	0.817
PANSS negative symptom	-0.041	0.747	-0.086	0.494	0.028	0.826	-0.03	0.813	0.139	0.271	-0.129	0.304	0.087	0.492
General psychopatholog symptom	0.064	0.612	-0.129	0.304	0.024	0.852	0.005	0.971	0.057	0.654	-0.198	0.115	-0.081	0.523
PANSS total score	0.016	0.9	-0.073	0.565	0.002	0.986	-0.055	0.662	0.076	0.545	-0.113	0.368	-0.034	0.789
BACS total score	0.083	0.511	0.209	0.094	0.101	0.423	0.079	0.532	0.029	0.819	0.216	0.084	-0.065	0.606
Verbal memory	0.007	0.954	0.228	0.067	0.033	0.792	-0.033	0.797	0.036	0.775	0.148	0.239	0.052	0.683
Working memory	0.02	0.875	0.083	0.511	0.042	0.739	0.082	0.518	-0.028	0.827	0.099	0.433	-0.199	0.112
Verbal fluency	0.081	0.519	0.074	0.558	0.111	0.379	0.103	0.415	-0.05	0.693	0.089	0.483	-0.157	0.213
Executive functioning	0.095	0.453	0.1	0.429	0.065	0.608	0.107	0.394	-0.107	0.396	0.041	0.744	-0.071	0.574
Attention and processing speed	0.187	0.136	0.194	0.122	0.261	0.035^*^	0.154	0.22	-0.058	0.644	0.222	0.076	-0.042	0.739
Motor speed	0.088	0.487	0.104	0.411	-0.015	0.904	0.03	0.811	0.114	0.365	0.136	0.281	0.05	0.694

PANSS, positive and negative syndrome scale; BACS, brief assessment of cognition in schizophrenia; CPZ,chlorpromazin; L, left; R, right; Lingual_R, the right lingual gyrus; Caudate_R, the right caudate nucleus; Lingual_L, the left lingual gyrus; Occipital_Mid_R, the right middle occipital gyrus; Frontal_Sup_Medial_R, the right medial superior frontal gyrus; Caudate_L, the left caudate nucleus; Postcentral_L, the left postcentral gyrus.*P<0.05;** P<0.01. The bold values indicates significant correlation.

## 4 Discussion

This is the first investigation of spontaneous brain activity differences, as measured by ALFF, among chronic schizophrenia patients undergoing long-term risperidone monotherapy, those treated with a risperidone–clozapine combination, and healthy individuals. Significant ALFF variations were identified across the bilateral lingual gyrus, bilateral caudate nucleus, right middle occipital gyrus, right medial superior frontal gyrus, and left postcentral gyrus compared to HCs. Notably, the RCT-SZ group exhibited reduced ALFF in the right caudate nucleus compared to the RT-SZ group. Furthermore, ALFF in the left lingual gyrus a trend of positive association with cognitive performance in attention and information processing speed among patients, suggesting a potential functional relevance. The study suggests that the right caudate nucleus may be a potential key brain region affected by the combination therapy of risperidone and clozapine in chronic schizophrenia. This finding provides preliminary clues for exploring the neuroimaging mechanisms related to this combination therapy and warrants further validation in future studies.

Both patient groups exhibited lowered ALFF in the bilateral lingual gyrus and elevated ALFF in the right caudate nucleus and right medial superior frontal gyrus relative to HCs. This pattern of results partially supports previous findings regarding abnormal brain activity in schizophrenia, but there are also notable differences, highlighting the potential heterogeneity of research results in this field. This study found that the reduction in bilateral lingual gyrus ALFF and the increase in right medial superior frontal gyrus ALFF were consistent with the results of a meta-analysis (37). Similarly, the finding of increased right caudate nucleus ALFF was consistent with the theoretical expectation of increased striatal functional activity (37, 38). However, the increased ALFF in the right medial superior frontal gyrus observed in this study does not fully correspond with the bilateral superior frontal gyri ALFF elevation reported in another meta-analysis (39) in terms of lateralization, and no significant ALFF changes in the caudate nucleus region were reported in that study, whereas this study clearly observed a significant increase in ALFF in the right caudate nucleus. These discrepancies may stem from the fact that this study specifically focused on chronic schizophrenia patients undergoing long-term treatment with specific antipsychotic medications, whereas the meta-analyses (37, 39) included multiple studies that typically encompassed patients on different treatment medications, at different treatment stages, and with varying disease durations. Additionally, the sensitivity to changes in specific small brain regions may be influenced by the heterogeneity of the original studies (e.g., scanning parameters, statistical thresholds, etc.). Importantly, our findings showed significantly increased ALFF in the caudate nucleus in both RT-SZ and RCT-SZ groups compared to HCs. As a core component of the striatum, which includes the caudate and putamen, the caudate nucleus is known to play a pivotal role in schizophrenia pathophysiology (40, 41). The striatal dopamine hypothesis posits that hyperactivity in the striatum contributes to the emergence of psychotic symptoms in schizophrenia (38). Our results lend support to this hypothesis, suggesting that heightened striatal activity may represent a key neurofunctional marker in chronic schizophrenia, regardless of treatment regimen. Moreover, the observed difference in right caudate activity between the RCT-SZ and RT-SZ groups may highlight the potential impact of combination therapy on striatal function, offering novel information regarding the neural mechanisms with a basis for differential antipsychotic treatment effects.

Our analysis revealed significantly reduced ALFF in the right caudate nucleus in RCT-SZ group compared to the RT-SZ group. Alterations in striatal functional activity are well-documented imaging markers in schizophrenia and have been strongly linked to antipsychotic treatment responsiveness (42). The core therapeutic mechanism of antipsychotic drugs involves antagonism of dopamine D2 receptors (DRD2) (43), and the caudate nucleus is a brain region highly enriched in DRD2 (44). Extensive evidence suggests that the occupancy rate of DRD2 in the striatum, particularly the caudate nucleus, is directly correlated with the clinical efficacy of antipsychotic drugs (45–47). Research has found that short-term treatment with the DRD2 antagonist risperidone can lead to increased ALFF in the caudate nucleus (48). This is thought to be due to the drug’s acute antagonism of dopamine-induced vasoconstriction, thereby increasing local cerebral blood flow and BOLD signal (49). However, long-term DRD2 antagonism exhibits different neuroadaptive effects. Animal model studies have shown that sustained DRD2 blockade can lead to inhibition of striatal dopaminergic neuron discharge activity, and even depolarization blockade, ultimately reducing the overall excitability of the striatum (50, 51). Importantly, the binding strength of DRD2 antagonists has been shown to be negatively correlated with BOLD signal amplitude, with stronger receptor binding associated with lower signal intensity (52). Based on previous research findings, we speculate that short-term antagonism may temporarily increase ALFF due to vascular effects, while long-term antagonism may reduce ALFF by inducing neuronal activity inhibition. Although risperidone is a potent DRD2 antagonist, clozapine has relatively low affinity for DRD2 and a shorter duration of action. However, the combined use of two drugs with DRD2 antagonistic activity may increase the overall occupancy or duration of action of DRD2 receptors in the caudate nucleus (53). Additionally, clozapine has a complex receptor action spectrum (54) and can indirectly influence the excitability of striatal neurons by regulating cortico-striatal glutamatergic input (55). Therefore, the study identified significantly decreased ALFF in the right caudate of the RCT-SZ group compared to the RT-SZ group.This may reflect that combination therapy may have led to a more sustained or enhanced antagonistic state of the caudate nucleus DRD2 system, thereby triggering more significant adaptive neuronal activity inhibition. The multi-target effects of clozapine may also be involved. However, there is currently a lack of direct evidence measuring DRD2 occupancy in the caudate nucleus of both groups to confirm this hypothesis. Future studies should integrate multi-modal imaging to directly assess the relationship between receptor occupancy and BOLD signals, and longitudinally track the dynamic evolution of striatal functional activity under different treatment regimens to more precisely elucidate the underlying mechanisms.

Although this association did not reach statistical significance after Bonferroni correction, there was a positive correlation trend between ALFF in the left lingual gyrus of patients with chronic schizophrenia and measures of attention and processing speed, which is consistent with previous studies reporting the involvement of the lingual gyrus in cognitive processing (56). The lingual gyrus, located in the occipital lobe, is closely involved in visual memory, attentional control, and broader cognitive functioning (57). This suggests that reduced spontaneous neural activity in the lingual gyrus may underlie attentional deficits and slower information processing in schizophrenia. In addition, ALFF in the bilateral lingual gyrus and left caudate nucleus showed a negative correlation trend with disease duration. Prior findings have reported diminished ALFF in the lingual gyrus (37) and elevated ALFF in the left caudate nucleus in schizophrenia (58). Our results suggest that tongue activity may decrease with disease duration, while increased ALFF in the left caudate nucleus may persist throughout the disease course. Although the corrected P-value did not reach the significance threshold, the r-value was close to the medium effect size, suggesting that these region-specific ALFF changes may have exploratory value as biomarkers of disease progression (59). It is worth noting that although the correlation between ALFF in the right caudate nucleus and BACS total score and verbal memory was not significant, a consistent positive trend was observed. Furthermore, earlier research has found a significant correlation between ALFF values in the bilateral caudate nucleus of schizophrenia patients and cognitive impairment (20). These results suggest that caudate nucleus neural activity may be potentially linked to cognitive function, and further validation with a larger sample size is needed in the future. The correlation results of this study collectively suggest that although the current evidence is insufficient to confirm a statistically significant association between ALFF in specific brain regions and clinical indicators, the observed trend effects provide important directions for future research.

## 5 Limitations

Several limitations are present. The primary limitation of this study is its non-randomized observational design with a small sample size, which significantly increases the risk of selection bias. Patients receiving combination therapy may represent a treatment-resistant subgroup, and systematic differences in their baseline characteristics may confound the interpretation of neuroimaging results. However, this study is positioned as exploratory work, with the core objective of providing a testable biomarker hypothesis for future randomized controlled trials. Second, this study cannot be used to effectively draw causal inferences given its cross-sectional design. Nevertheless, by carefully matching groups on demographic and clinical variables, including illness duration, PANSS scores, and BACS assessments, and applying appropriate covariate adjustments, we aimed to minimize the influence of confounding factors. Third, the significant differences in CPZ equivalent doses between groups primarily stem from the inherent clinical characteristics of the combination therapy strategy and should not be eliminated through statistical correction to avoid overcorrection bias. When sample sizes are limited, adding covariates reduces model degrees of freedom; moreover, CPZ equivalent doses are estimated based on receptor binding and cannot accurately reflect whole-brain effects, thereby limiting their reliability as covariates. Therefore, the observed differences in brain function may be influenced by dose and patient heterogeneity. Future studies should adopt a prospective design with fixed doses and strict intergroup matching to effectively distinguish between strategy effects and dose effects. Finally, the absence of a group comprising long-term untreated chronic schizophrenia patients restricts our ability to disentangle the specific contributions of disease duration from those of prolonged medication exposure. However, the main focus of this study was comparing the neurofunctional impacts of two long-term treatment strategies rather than to isolate the effects of duration versus medication. Furthermore, in our clinical context, patients with chronic schizophrenia are typically treated consistently, with long untreated durations being extremely rare. Importantly, no correlation was evident between illness duration and right caudate ALFF in our data, which may indirectly reduce concerns that disease duration alone accounts for the observed group differences. Nonetheless, future longitudinal or multi-center studies will be essential for validating and extending these findings.

## 6 Conclusion

As an exploratory study, the results indicate that chronic schizophrenia patients treated with a combination of risperidone and clozapine exhibit significantly lower ALFF in the right caudate nucleus compared to those on risperidone monotherapy. In addition, higher ALFF in the left lingual gyrus tended to be associated with better attention and information processing. These results suggest that different long-term drug treatment strategies may influence spontaneous neural activity patterns and provide preliminary neuroimaging clues to the potential mechanisms of combination therapy in schizophrenia, which warrants further validation in future studies.

## Data Availability

The raw data supporting the conclusions of this article will be made available by the authors, without undue reservation.
